# The many faces of autoimmune-mediated melanocyte destruction in melanoma

**DOI:** 10.3389/fimmu.2024.1417273

**Published:** 2024-10-03

**Authors:** Loredana Ungureanu, Alina Florentina Vasilovici, Salomea-Ruth Halmágyi, Ioana Irina Trufin, Adina Patricia Apostu, Simona Corina Şenilă

**Affiliations:** ^1^ Department of Dermatology, Iuliu Hatieganu University of Medicine and Pharmacy, Cluj-Napoca, Romania; ^2^ Department of Dermatology, Emergency County Hospital, Cluj-Napoca, Romania; ^3^ Department of Dermatology, Clinical Hospital of Infectious Diseases, Cluj-Napoca, Romania

**Keywords:** melanoma, autoimmunity, melanocytes, vitiligo, melanoma-associated leukoderma, immunotherapy, targeted therapy

## Abstract

Melanoma is the most severe form of skin cancer with an incidence that is increasing all over the world. Melanoma cells derive from normal melanocytes and share different melanocyte-specific antigens, the same antigens against which an immune reaction develops in vitiligo, a skin disease characterized by autoimmune-mediated melanocyte destruction. The purpose of this review is to present the autoimmune-mediated melanocyte destruction associated with melanoma development, progression and treatment. Patients with vitiligo seem to have a lower chance of developing melanoma. On the other hand, patients with melanoma can develop depigmented lesions even at distant sites from the primary tumor, defined as melanoma-associated leukoderma (MAL). Drug-associated leukoderma (DAL) was also described in melanoma patients treated with immunotherapy or targeted therapy and it seems to be a favorable prognostic factor. Clinically, MAL and DAL can be diagnosed as vitiligo and there are few differences between these three entities. In this review, the incidence of DAL in melanoma patients treated with different therapies was researched in the literature and patient outcome was recorded, with studies showing a prolonged disease-free survival in melanoma patients with DAL, treated with immune checkpoint inhibitors. Further studies are however needed to understand the dynamics of autoimmune-mediated melanocyte destruction.

## 1 Introduction

Melanoma, the malignant tumor derived from melanocytes, is the most threatening form of skin tumors, with a continuously increasing incidence all over the world (1, 2). Although it represents less than 5% of all skin cancers, melanoma is responsible for the greatest number of skin cancer-related deaths worldwide (3). Due to the high mutational burden, melanoma is an immunogenic tumor, which is highlighted by its ability to undergo spontaneous regression and also immunotherapy-induced regression (3–7). Cancer immunity relies on the recognition of antigens by the host’s immune system. A large number of melanoma antigens that are recognized by the immune system have been described, more than for any other cancer (5). Melanoma-specific cytotoxic T lymphocytes have been observed both in the skin that surrounds the tumor and in the patient’s blood. Melanoma antigens that can be recognized by T-cells are classified as follows: (a) tumor-specific neo-antigens derived from DNA mutations, (b) cancer-germline antigens, which are tumor-specific shared antigens (3), melanocyte-specific differentiation antigens (e.g. gp100, MART-1, tyrosinase, TRP-1, TRP-2) and (4) other antigens such as viral antigens or overexpressed proteins (8). Melanoma cells originate from normal melanocytes, which explain why these two types of cells share many melanocyte antigens that are expressed by melanocytes in the skin of healthy donors, in the healthy skin of vitiligo patients and by tumor cells in melanoma patients (9). Melanocyte-specific antigens are proteins, and peptides that derive from them can be recognized by antigen-specific CD8+ T cells, and high levels of melanocyte-specific CD8+ T cells can be detected in the blood and among tumor-infiltrating lymphocytes in melanoma, as well as in patients with vitiligo (9).

Vitiligo is an autoimmune skin disease characterized by circumscribed or generalized depigmentation of the skin and mucosa, as a result of autoimmune-mediated melanocyte destruction (10). The importance of autoimmunity is highlighted by the frequent association with other autoimmune disorders and especially by the presence of autoantibodies against melanocyte differentiation antigens in the blood of a significantly higher percentage of patients with vitiligo compared with healthy individuals (5). Moreover, circulating melanocyte-specific CD8+ T cells, as well as infiltrates of T cells at the margins of active lesions have been described in most patients (11).

The present review aims at providing an overview of autoimmune-mediated melanocyte destruction that can appear in association with melanoma, from the risk of developing melanoma to melanoma prognosis and response to therapy. In order to assess the incidence of patients treated with different therapies that developed MAL, we researched the literature (PubMed) using these terms: “vitiligo melanoma treatment”, “vitiligo melanoma immunotherapy”, “vitiligo melanoma anti-PD1 therapy”. Only articles in English were selected and duplicates were excluded.

## 2 Autoimmune-mediated melanocyte destruction and the risk of developing melanoma

Due to the absence of melanin in depigmented lesions and secondary to incidental and therapeutic UV light exposure, concerns were raised about the development of skin cancer, especially melanoma, in vitiligo patients. However, genetic studies suggested a lower susceptibility of developing melanoma in patients with vitiligo (12). A genome-wide association study in patients with vitiligo showed significant associations between vitiligo and several genes that regulate immunity (13). Vitiligo was associated with a polymorphism in the gene that codes the main enzyme involved in melanin synthesis, tyrosinase (TYR gene). Interestingly, the TYR allele that confers risk for vitiligo is protective against melanoma, suggesting that strong anti-tyrosinase expression protects vitiligo patients against melanoma (13). Moreover, in a meta-analysis conducted by Liu et al. human-leukocyte antigen – A2 proved to be the protective allele against melanoma development, and at the same time the risk allele for vitiligo development (14). According to Wu et al. the inverse relationship might indicate that different or opposed biological pathways mediate vitiligo and skin cancer, meaning that an enhanced immune activity against melanoma can appear in vitiligo (12).

Epidemiological studies showed inconsistent results. Lindelof et al. found that vitiligo patients have a lower risk of developing melanoma (15). Teulings et al. found a threefold lower probability of developing melanoma in vitiligo patients in a retrospective comparative cohort study (16). In a study that included 10.040 patients with vitiligo, Paradisi et al. showed that they were 4 time less likely to develop melanoma compared with controls, the difference being highly statistically significant (17). Jorgensen et al. conducted a population-based study including 2,339 subjects with vitiligo and 23,293 controls, but their study showed no significant difference in what concerns the risk of cutaneous melanoma (18). Kim et al. showed that in Korean vitiligo population, the risk of melanoma is increased; however, skin cancer incidence in Korean patients was much lower than in their white counterparts (19). The latest and largest European study that included 15,156 vitiligo cases showed that vitiligo is associated with a substantially lower risk of new-onset skin cancer, for both melanoma and non-melanoma skin cancer (20).

Multiple mechanisms were proposed to explain the negative association between vitiligo and melanoma, including the use of sunscreens, the role of anti-melanocyte immune response in vitiligo, the absence of melanocytes in vitiligo lesions, and the overproduction of proinflammatory cytokines that stimulates the production of superoxide dismutase and glutathione peroxidase, thus reducing the risk of melanoma (21, 22).

## 3 Spontaneous autoimmune-mediated melanocyte destruction in melanoma

The development of depigmented lesions in melanoma patients has been reported for the first time more than 45 years ago, and confirmed by case reports, small patient series and a few studies (15, 23–31). Four patterns of depigmented lesions have been described in melanoma: (a) areas of depigmentation confined to the primary lesion, suggesting spontaneous regression, (b) areas of depigmentation around the primary tumor, (c) areas of depigmentation around benign melanocytic nevi (halo phenomenon), and (d) widespread hypomelanosis occurring at distant sites from the primary tumor (melanoma associated leukoderma - MAL) (24). The four types of depigmentation are not mutually exclusive and can occur simultaneously (24).

MAL occurs spontaneously in a fraction of melanoma patients, before or after the detection of melanoma (32). Quaglino et al. conducted a prospective cohort study on 2,954 patients of all stages with melanoma and found a 2.8% prevalence of melanoma-associated vitiligo, higher than the prevalence of vitiligo in the general population (33). The authors also showed that melanoma patients with MAL have a higher frequency of immune-mediated manifestations than melanoma patients without MAL, although MAL is less likely to be associated with autoimmune conditions than vitiligo (33). Quaglino et al. showed that depigmented lesions can appear before melanoma diagnosis, after surgical excision, after locoregional metastases, or after distant metastases (33).

The differences and similarities between MAL and vitiligo regarding clinical presentation are not very well defined, the literature being contradictory (**Table 1**). Studies show that the age at onset was significantly higher in patients with MAL than in vitiligo patients (32). A positive family history of vitiligo is reported in some studies, while in others a positive history was absent in all cases (24, 32, 34). Quaglino et al. found a symmetrical, bilateral distribution in the majority of patients, similar to that in vitiligo (33). Hartmann et al. found a symmetrical distribution pattern in 75% of MAL patients and no correlation between the distribution of the hypopigmentation and the location of the primary tumor (34). On the other hand, Koh et al. and Nordlund et al. showed that MAL is mostly characterized by hypopigmented macules with irregularly shaped border and confetti-like appearance, different from well-demarcated white macules in vitiligo (24, 25). Moreover, equal distribution among men and women, distribution on sun-exposed areas and multiple flecked depigmented macules were also described as clinical features that are distinct from vitiligo (35, 36).

**Table 1:** Clinical aspect of vitiligo, MAL and DAL.

Clinical presentation	Vitiligo (non-segmental)	Spontaneous MAL	DAL
**Onset**	Average age – 20 years	>50 years	Mean onset delay of 30 weeks after therapy initiation
**Clinical aspect**	Well-demarcated white macules/patches Symmetrical distribution Presence of the Koebner phenomenon	Even distribution in both men and women Round-patchy confetti-like lesions Symmetrical, bilateral distribution Absence of the Koebner phenomenon	Multiple flecked lesions Absence of the Koebner phenomenon
**Localization**	- face - dorsal hands - nipples - axillae - inguinal and anogenital	- sun-exposed areas - trunk - extremities - face	- predilection sites for non-segmental vitiligo - sun-exposed areas (face, chest, and hands)
**Autoimmune disease association**	yes	no	no
**Evolution**	Insidious/unpredictable	Rapidly progressive	Repigmentation possible after ICI discontinuation
**Treatment response**	variable	Refractory to topical steroids and UV therapy	No therapy is indicated initially, in order not to interfere with the response to immunotherapy
**Halo phenomena**	Depigmentation around nevi	Depigmentation around Melanoma Nevi Cutaneous metastases Scars	Depigmentation around Melanoma site Nevi Cutaneous metastases
**Melanocyte-specific T cells against MART-1, gp100 and tyrosinase**	Low levels in stable vitiligo Higher levels in active vitiligo	Low levels	–
**Autoantibodies** **gp100 and tyrosinase antibodies** **MART-1 antibodies**	Yes No	Yes Yes	Yes Yes
**CXCL 10 in the blood**	Low levels in stable vitiligo Higher levels in active vitiligo		High levels

Lommerts et al. conducted a study to assess whether experts in the field can distinguish between MAL and vitiligo, and to assess if discriminative features can be identified. The authors showed that even experts cannot clearly differentiate between MAL and vitiligo only based on photographs, no significant differences being identified in the clinical presentation. As a consequence, a total body skin examination should be performed in all patients with seemingly typical vitiligo, especially if the age of onset is higher (32).

Teulings et al. retrospectively analyzed the clinical presentation, type of depigmentation and disease course of patients who developed MAL within one year before the detection of a primary melanoma or within 3 years before the detection of melanoma metastases with an unknown primary tumor, and identified seven patients initially diagnosed with non-segmental vitiligo as having MAL (37). They were older Caucasian patients, with sudden onset of rapidly progressive skin depigmentation on non-typical vitiligo predilection sites with median to sharp demarcations and often consisting of round patchy confetti-like lesions, mostly symmetrically scattered over the trunk, extremities and/or face; lesions were generally refractory to topical steroids and UV therapy (37). The authors emphasize that special attention should be given to older Caucasian patients presenting with late onset, rapidly progressing atypical vitiligo-like depigmentation refractory to standard treatment (37).

In some cases, the appearance of depigmented lesions revealed a regressing melanoma (31). Moreover, two cases of melanoma developing within a depigmented patch were recently described, suggesting that clinicians should be cautious in the presence of a new, solitary, vitiligo-like patch in a patient with no risk factors for vitiligo (38).

Hartmann et al. failed to show histological and immunohistological differences between MAL and vitiligo. No differences were described regarding the number of epidermal melanocytes and dendritic cells or the inflammatory infiltrate in terms of characteristics or composition (34).

Multiple studies showed that MAL is associated with a favorable prognosis (6, 33, 37, 39). Quaglino et al. found no statistically significant difference regarding disease free survival (DFS) in stage I-II patients based on the presence of MAL. On the other hand, MAL was associated with a significantly higher overall survival and DFS in stage III-IV melanoma patients, independent of treatment, with no difference in survival according to the time onset of MAL (33).

It is hypothesized that MAL is caused by anti-melanoma immunity targeting normal and malignant melanocytes, due to the presence of melanocyte differentiation antigens on both cells. Teulings at al. studied the immunological differences in patients with MAL and vitiligo. They found specific T cells against MART-1, gp100 and tyrosinase in the blood of both patients with MAL and vitiligo, although low levels were detected in MAL and stable vitiligo, while more melanocyte specific T cells were found in active vitiligo. Autoantibodies against gp100 and tyrosinase were found in both diseases, but MART-1 antibodies were only present in patients with MAL (40).

Palermo et al. found that melanocyte specific T cell response differs qualitatively, not quantitatively in melanoma and vitiligo. They did not find significant differences in the precursor frequencies of Melan-A-specific cytotoxic T lymphocytes, nor in their status of activation. However, they documented a higher receptor affinity of melanocyte T cells from vitiligo. Moreover, only T cells from vitiligo patients were capable of efficient TCR downregulation and IFN-γ production in response to HLA-matched melanoma cells, emphasizing that the receptor affinity difference is physiologically relevant (41).

Vitiligo patients have an increased frequency of halo nevi, while multiple halo nevi might predispose to the onset of vitiligo (42). Halo phenomena have been described in melanoma patients, not only around melanoma and benign nevi, but also around cutaneous metastases and scars in patients with MAL, but its significance is not fully understood (33, 43, 44).

## 4 Therapy-induced autoimmune-mediated melanocyte destruction in melanoma

### 4.1 Immunotherapy-induced autoimmune-mediated melanocyte destruction in melanoma

Teulings et al. conducted a systematic review of 137 studies including 5,737 patients with stage III to IV melanoma treated with immunotherapy between 1995 and 2013, and found a pooled incidence of drug-associated leukoderma (DAL) of 3.4% (45). Their review suggested that patients with melanoma that develop DAL have a two-fold decreased risk of disease progression and a four-fold decreased risk of death compared with patients without DAL, suggesting that DAL is a clinical marker for effective anti-melanoma immunity and clinical outcome (45). Various immunotherapies were studied in the review coordinated by Teulings, including general stimulation with interferon-α (IFN- α) or interleukin-2 (IL-2), a modified oncolytic virus, and immune check-point inhibitors, the last being the standard immunotherapy for melanoma nowadays (45).

#### 4.1.1 Immune checkpoint inhibitors

ICI, namely anti-cytotoxic T-lymphocytes antigen-4 (CTLA-4) and programmed cell death (PD)-1 inhibitors, act by upregulating the anti-tumor immune response, enhancing T-cell activation (46).

DAL can occur in up to 2-25% of patients treated with ICI for melanoma, generally occurring at higher rates after anti-PD-1 therapy compared with CTLA-4 inhibition, with a mean onset delay of 30 weeks after therapy initiation (32, 37, 44, 45, 47, 48). The appearance of DAL after ICI is significantly associated with favorable prognosis in multiple studies (45, 48–50). In patients treated with anti-PD-1 agents, DAL is independently associated with a better response rate and better overall survival (49, 51).

Larsabal et al. found that DAL occurring in patients receiving anti-PD-1 therapies is clinically different from vitiligo. Accordingly, no family history of vitiligo, thyroiditis or other autoimmune disorders is reported, and the Koebner phenomenon is absent. Moreover, depigmented lesions can occur on predilection sites for non-segmental vitiligo, but also on sun-exposed areas (face, chest and hands) (**Table 1**) (52). Lommerts et al. demonstrated, however, the lack of clear discriminative features between DAL and non-segmental vitiligo, both clinically and histologically (32).

Depigmented lesions were also described around primary melanoma sites and around cutaneous metastases, and the concomitant presence of leucotrichia, as well as halo nevi, was also documented (32, 49). Rarely, DAL can be associated with autoimmune bilateral diffuse granulomatous uveitis during or after ICI (53). After the discontinuation of ICI, repigmentation can occur, but it usually resides (46). Matsuya et al. tried to evaluate the correlation between DAL dynamics and clinical efficacy of anti-PD-1 antibodies. They found that DAL expansion and grade 2 DAL (depigmented lesions affecting more than 10% of body surface) showed no improvement in treatment response, but were associated with prolonged progression-free survival and a trend for prolonged overall survival (54).

Hua et al. performed skin biopsies from depigmented lesions developed during pembrolizumab therapy and observed a dermal inflammatory infiltrate composed of T cells and the disappearance of melanocytes (49). Freeman-Keller proposed that PD-1 mediates tolerance to melanosomal proteins, and inhibition of PD-1 activity leads to autoimmune depigmentation (55). Le Gal et al. demonstrated that CD8+ T-cells are the main effector cells that recognize melanocyte differentiation antigens, being present in both tumor and DAL tissue (56). Schumacher et al. showed that neoantigen-specific T-cells are important active ingredients for successful immunotherapy (57), however in patients with low neoantigens, the immunity against melanocyte differentiation antigens is relevant in rescuing suboptimal immune activation (58). Lo et al. found that patients with low melanoma neoantigen burden that responded to ICI had tumors with higher expression of pigmentation-related genes. Moreover, expansion of peripheral blood CD8+ T cells against melanocyte specific antigens was observed only in patients who responded to ICI (59). Altogether, patients that respond to ICI despite low neoantigen load have an expansion of CD8+ T cell response against melanocyte specific antigens, which can also attack normal melanocytes leading to DAL, while responding patients that do not develop DAL may have effective neo-antigen immunity (46, 58).

Carbone et al. analyzed T cell subsets from the peripheral blood of metastatic melanoma patients undergoing anti-PD-1 therapy and skin biopsies collected from those who developed DAL, and sequenced T cell receptor (TCR) cells derived from biopsies of DAL and primary melanoma of the same patient (59). DAL development was associated with blood reduction of CD8+ mucosal-associated invariant T (MAIT), T helper (h) 17, natural killer (NK) CD56^bright^, and T regulatory (T-reg) cells. A high amount of IL17-A expressing cells in DAL biopsies was also documented, suggesting a possible migration of Th17 cells from the blood in depigmented lesions. Interestingly, in most of the cases, they found different TCR sequences between DAL and primary tumor, but shared TCR sequences between DAL and metastatic tissue of the same patient (59). They concluded that T-cell response against normal melanocytes, which leads to DAL, is mediated by T-cell clones targeting metastatic tissues, rather than reactivation of specific T-cell clones infiltrating primary melanoma. Altogether, anti-PD-1 therapy seems to induce a *de novo* immune response, triggered by the presence of metastatic tissue (59).

There are conflicting reports regarding the humoral response in ICI-induced DAL. Development of antibodies can occur secondarily to T-cell melanoma cell destruction and antigen release. As mentioned before, Teulings et al. showed that autoantibodies directed against gp100 and tyrosinase were found in both vitiligo and DAL, but MART-1 antibodies were only present in patients with DAL (40). Moreover, Larsabal et al. found higher levels of the chemokine CXCL10 in the blood of melanoma patients developing DAL after ICI compared with vitiligo patients and healthy controls (52). However, higher amounts of CXCL10 were found in active vitiligo, suggesting that reported differences between ICI-induced DAL and active vitiligo could be less significant (60).

Although DAL is considered an adverse effect of ICI therapy, treatment can be continued as DAL is associated with better survival and better response rates. Local or systemic immunosuppression is not recommended, as it may hypothetically decrease the response to ICI therapy. However, local treatment can be considered if DAL persists after extended discontinuation of immunotherapy (46).

The incidence of DAL in melanoma patients using different therapies is summarized in **Table 2**.

**Table 2:** Incidence of DAL in melanoma patients treated with different therapies.

Study	Number of patients	Diagnosis	Pembrolizumab	Nivolumab		Ipilimumab		Ipilimumab + Nivolumab Combination	Outcome
Rosenberg and White 1996 (61)	74	Stage IV melanoma and Stage IV Renal Cell Carcinoma (RCC)						(high dose) 15% (11 of 74) patient with melanoma overall 26% (11 of 43) patient with response to immunotherapy	MAV was not observed in patients unresponsive to therapy; moreover, it did not occur in patients treated for RCC
Hamid et al., 2013 (62)	135	Stage III/IV melanoma	9% (lambrolizumab)	NR					
Potsow et al., 2015 (63)	142	Stage III/IV melanoma				4 (8.7%)		10 (10.6%)	NR
Sanlorenzo et al., 2015 (64)	66	Stage III-IV melanoma,	7 (8%)						
Ribas et al., 2015 (65)	180 pembrolizumab 2 mg/kg 181 pembrolizumab 10 mg/kg	Stage III/IV melanoma	10 (6%) pembrolizumab 2mg/kg 9 (5%) pembrolizumab 10 mg/kg						NR
Robert et al., 2015 (66)	834	Stage III-IV melanoma	9% q2w 11.2% q3w						
Teulings et al., 2015 (40)	5737	Stage III-IV melanoma	2.0% (ipilimumab or tremelimumab) or anti-PD1 antibodies (nivolumab or lambrolizumab)						
Robert et al., 2015 (67)	418	Stage IV melanoma		10.7%					NR
Hwang et al., 2016 (68)	82	Stage IV melanoma	12 (14.6%)					NR	
Goldinger et al., 2016 (69)	68	Stage IV melanoma	10 (15%)					NR	
Hua et al. 2016 (49)	67	Stage IV melanoma	17 (25%)						
Long et al., 2017 (70)	153	Stage III/IV melanoma						30 (20%) pembrolizumab + ipilimumab	NR
Nakamura et al., 2017 (50)	35	Stage III-IV melanoma	–	9 (25.7%)					The objective response rate was higher in patients who developed vitiligo compared to those who didn’t develop vitiligo (44.4% vs. 7.7%)
Weber et al., 2017 (71)	576	Stage III/IV melanoma		7.8%					NR
Wen et al., 2017 (72)	52: 14 (ipi), 28 (pembro), 10 (pembro+ ipi)	Stage III/IV melanoma	5 (18%)			0		2 (20%) pembrolizumab + ipilimumab	NR
Yamakazi et al., 2017 (73)	42	Stage III/IV melanoma	3 (7.1%)						NR
Yamakazi et al., 2017 (74)	24	Stage III/IV melanoma		9 (37.5%)					Patients with vitiligo showed a tendency for better survival
Dika et al. 2017 (75)	41	Stage IV melanoma							
Eggermont et al., 2018 (76)	509	Stage III melanoma	24 (4.7%)						NR
Larkin et al., 2018 (77)	272	Stage III/IV melanoma		29 (11%)					NR
Indini et al., 2018 (48)	173	Stage IV melanoma	8 (4%) - immunotherapy						The occurrence of vitiligo was associated with a trend toward non-significantly improved overall survival
Hwang et al., 2019 (78)	25	Stage IIIC/IV melanoma						7/25 (28%) (ipilimumab+ pembrolizumab)	There was no statistically significant association between the development of vitiligo and treatment response
Quach et al., 2019 (79)	318	Stage III-IV melanoma						120 (38%)	Superior response rate associated with the development of vitiligo
Matsuya et al., 2020 (54)	29	Stage III/IV melanoma	6 (20.7%)	23 (79.3%)					Patients who advanced to grade 2 vitiligo showed a slight tendency toward better treatment response
Nakano et al., 2020 (80)	128	Stage III/IV melanoma		25 (19.53%)					NR
Nardin et al., 2020 (51)	111	Stage III-IV melanoma	15 (13.5%) immunotherapy						Vitiligo development was associated with greater overall survival and confirmed in landmark progression-free survival.
Bottlaender et al., 2020 (81)	189	Stage IV melanoma	16 (8.5%)						Better OS in patients with vitiligo
Zhao et al., 2020 (82)	93	Stage IV melanoma	15 (16.1%)						Better clinical outcome in patients with vitiligo
Wu et al., 2020 (83)	49	Stage III/IV melanoma	2 (4.08%)						
Dousset et al., 2021 (84)	457	Stage III/IV melanoma	85 (estimated cumulative incidence of 18.6%) – 60% of patients received pembrolizumab and 40% received nivolumab					The presence of vitiligo was associated with significantly improved overall survival and progression-free survival	
Guida et al. 2021 (85)	148	Stage IV melanoma	83 (56%)			47 (32%)		18 (12%)	52% progression free after 3 years
Patel et al., 2021 (86)	81	Stage III/IV melanoma	11 (12%) pooled incidences of different therapeutic approaches including pembrolizumab, nivolumab, and ipilimumab						NR
Yamazaki et al., 2021 (87)	124	Stage III/IV melanoma			14 (11.29%)				Longer OS
Verkhovskaia et al., 2021 (88)	280	Stage III/IV melanoma	43 (15.4%)						Vitiligo – marker of favorable outcome
Shreberk-Hassidim et al., 2022 (89)	95	Stage IV melanoma	17 (17.9%)- anti PD1						NR
Nikolaou et al., 2022 (90)	199	NR	37 patients (18.6%)						
Medri et al., 2023 (91)	182	Stage IV melanoma	Ipilimumab + Nivolumab+ Pembrolizumab		11.5% (21 patients) developed vitiligo-like lesions				
L’Orphelin et al., 2023 (92)	120	Stage IV melanoma	Ipilimumab + Nivolumab+ Pembrolizumab						23 vitiligo patients (19%) 3 patients- complete response* 6 patients- partial response* *at 6 months
Zhang et al., 2023 (93)	241	Stage III/IV melanoma	17 (7.1%)						
Asher et al., 2024 (94)	415	Stage III/IV melanoma	48 (11.6%)						

NR, Not reported.

Taking into account that not all melanoma patients respond to ICI, there is an increased need to identify some biomarkers that could predict treatment response and further research on the significance of DAL is also required to help determine its predictive value.

### 4.2 Targeted therapy-induced autoimmune-mediated melanocyte destruction in melanoma

Recently, DAL has also been described in patients treated with targeted therapies, being associated with better prognosis (95, 96). Ramondetta et al. hypothesized that targeted therapy-induced DAL can be explained by the effect on immune system cells related to the blockage of cancer cells by BRAF and MEK (95). Clinically, in contrast to classical vitiligo, which mainly involves the genitalia, wrists and perioral region, drug-induced DAL is localized on sun-exposed areas (face, neck, hands and arms). In the same study, the authors tried to classify drug-induced leukoderma according to the European Guidelines for the management of vitiligo, and found that the most frequent subtype is the non-segmental form (71.4%), particularly the generalized (40%) and acrofacial (40%) forms. In contrast, the distribution of spontaneous MAL was non-segmental, with a prevalence of the focal form (96).

## 5 Conclusions

Autoimmune-mediated melanocyte destruction in melanoma has many faces, starting with disease predisposition and continuing with diagnosis, prognosis and treatment response. Further studies are needed in order to better characterize the clinical picture, the differences and similarities between MAL and vitiligo, the differences and similarities between MAL and DAL. Moreover, understanding the mechanism of concomitant autoimmune destruction of normal and malignant melanocytes could help select patients with an increased likelihood of therapeutic response, especially after ICI. Treatment options for depigmented lesions in DAL and the risk of decreasing the response to melanoma therapy in these patients also require further studies.
